# Continuity of chronic predation risk determines changes in prey physiology

**DOI:** 10.1038/s41598-020-64000-9

**Published:** 2020-04-24

**Authors:** Łukasz Jermacz, Hanna Kletkiewicz, Anna Nowakowska, Anna Dzierżyńska-Białończyk, Maciej Klimiuk, Jarosław Kobak

**Affiliations:** 1 Nicolaus Copernicus University, Faculty of Biology and Veterinary Sciences, Department of Invertebrate Zoology and Parasitology, Lwowska 1, 87-100 Toruń, Poland; 2 Nicolaus Copernicus University, Faculty of Biology and Veterinary Sciences, Department of Ecology and Biogeography, Lwowska 1, 87-100 Toruń, Poland; 3 Nicolaus Copernicus University, Faculty of Biology and Veterinary Sciences, Department of Animal Physiology, Lwowska 1, 87-100 Toruń, Poland

**Keywords:** Ecology, Ecophysiology, Freshwater ecology, Invasive species

## Abstract

Prey reconfigure their physiology to avoid costs of prolonged predator pressure. However, these changes might not occur under periodic predation risk, with repeating acute phases. To test the effect of predation risk continuity on changes in prey physiology, we exposed amphipods: *Dikerogammarus villosus* and *Gammarus jazdzewskii* to periodic and constant predation cue. After one week, we measured: cellular defence systems: total antioxidant status (TAS), heat shock proteins (Hsp70); intracellular damage marker: lipid peroxidation (TBARS); condition index: glycogen concentration. Predator presence reduced TAS level in *G. jazdzewskii* independent of its continuity and in *D. villosus* after periodic exposure. Amphipods showed downregulation of Hsp70 when exposed to periodic (*D. villosus*) or constant (*G. jazdzewskii*) predation risk. Exposure to predators reduced TBARS level in *D. villosus* (irrespective of the continuity) and *G. jazdzewskii* (periodic exposure). Glycogen concentration in both species was not affected by predator presence. Thus, the continuity of the predator cue shaped prey physiology reconfiguration, optimizing costs of physiological adjustments under challenging conditions. Nevertheless, the lack of negative consequences of the prolonged exposure to the predator cue, whether constant or periodic, shows that amphipods can thrive under chronic predation risk, which is a constant part of the wild environment.

## Introduction

Predator pressure is a fundamental force driving evolution of prey species due to direct predation on the less adapted individuals (consumptive effect) as well as induction of costly defence mechanisms (non-consumptive effect), manifested as modifications of behaviour, physiology and/or life history^1–3^. To maximize the effectiveness of antipredator mechanisms, prey individuals relocate available resources, such as time and energy, into defence responses, limiting other activities, including food acquisition and reproduction^1,4,5^. Moreover, in contrast to direct predation, predator fear effect is perceptible by all threatened individuals, especially in aquatic systems, where chemical signals are the main source of information about predation risk^6^. Therefore, at the ecosystem scale, the impact of non-consumptive predator effects is comparable or even exceeds the effect of direct predation, shaping community structure and functioning to a great extent^1,7–9^.

During the recent years, a lot of attention has been focused on prolonged (chronic) effects of predation risk, especially on physiological responses driving behavioural and morphological changes responsible for preparation to defence reactions^10,11^. Previous studies showed that chronic exposure to predation risk caused diverse physiological modifications^10^. However, the obtained results seem inconclusive. For example, prey exposed to prolonged predation risk may exhibit either increased^12^ or reduced^13^ antioxidant defence. Prolonged predation risk is often interpreted as a factor responsible for chronic stress and reduction in prey fitness, including impairment of defence abilities^14^ as well as direct mortality^15,16^. On the other hand, prolonged exposure to predator signals has been demonstrated to induce reconfiguration of physiological processes necessary to compensate the costs of the defence reaction^17–19^. Moreover, some studies demonstrated that individuals pre-exposed to predation cues were better prepared to predator pressure than non-stressed ones^20,21^. Observed discrepancies could be caused by diverse factors, including different defence strategies of prey species^22^, specific compensatory mechanisms^18^ and food availability^12^. Here we postulate that the continuity of predation risk could be another crucial factor shaping reconfiguration of prey physiology.

Generally, in natural ecosystems, predator pressure is variable and unpredictable. However, in the aquatic environment, low concentration of predator kairomones is constantly present and defence reactions depend on the increase in the concentration of the chemical signal. Theoretically, a periodic predation risk offers time to regenerate and supplement energy resources, necessary to keep the defence response at the maximum effective level. Some studies demonstrated that most of the cost of the defence reaction, exhibited as the oxidative stress, was suffered during the initial exposure to the predation signal, while the subsequent prolonged and constant stimulation resulted in the reduction in oxidative damage despite the sustained increased metabolic rate^22^. One the other hand, it was demonstrated that a prolonged but periodic (i.e. interrupted by “safe” periods) predator pressure generated a significant oxidative damage^23^. Therefore, we hypothesized that the continuity of the predation risk is a key factor responsible for prey physiology reconfiguration resulting in the reduction of costs related to oxidative damage. To test this hypothesis, we checked whether total consequences of exposure to periodic predation risk (with the acute response phases repeated each time and separated by safe periods) could exceed those resulting from the constant predation risk (when prey can decrease their acute responses to overlapping exposure events). On the other hand, it is also possible that the effects of the constant exposure to the predator cue episodes exceed the compensatory abilities of prey, causing intracellular damage.

To test the above hypothesis, we exposed two species of freshwater gammarids (*Dikerogammarus villosus* and *Gammarus jazdzewskii*) to periodic (1 dose per day) and constant (8 doses per day) predation risk for one week. As the duration of the predator kairomone activity exceeds 3 h^24^, gammarids in the 8-dose treatment were under a constant impact of the predation cue, whereas the 1-dose treatment group was subject to a periodic exposure to the cue. To obtain a wide picture of changes in prey condition, we measured a range of physiological parameters informing of: (1) antioxidant defence potential (TAS: total antioxidant status), (2) intra-cellular damage as indicated by the level of lipid peroxidation (measured as the concentration of thiobarbituric acid reactive substances, TBARS), (3) concentration of a crucial but energetically costly part of the cellular defence system: heat shock proteins (Hsp70) and (4) concentration of the main energy storage material (glycogen).

## Material and methods

Gammarids originated from two locations: individuals of *D. villosus* were caught using trap substrata (artificial Christmas tree branches) in the nearshore zone of the Włocławek Reservoir (a dam reservoir on the lower River Vistula, Central Poland, N:52°37′03″, E:19°19′37″) while individuals of *G. jazdzewskii* were captured in the River Zielona Struga (a left tributary of the River Vistula, N:53°00′12″, E:18°27′39″) with a hand net. As a source of predator signals we used kairomones of the Eurasian perch (*Perca fluviatilis*) which is a natural predator for both gammarid species and co-occurs with them at both capture locations (personal observations). Fish were caught by electrofishing (device type EFGI 650, BSE Bretschneider Spezialelektronik, Germany) from the Włocławek Reservoir. Immediately after collection, all animals were transported to the laboratory and placed in 200-L stock tanks (each species separately) for one week of acclimation. The collection and use of fish were conducted under permit of the Local Committee for Ethics in Animal Research in Bydgoszcz, Poland, statement no 35/2013 from 12 December 2013. All procedures using fish were performed according to the European Union guidelines for the handling of laboratory animals (Directive 2010/63/UE). The bottom of the gammarid stock tanks was covered by pebbles and natural detritus collected from the same locations as gammarids. Water in each tank was constantly aerated and filtered. The water temperature was sustained by an air conditioner at 20 °C. During the acclimation period, gammarids were fed daily with a mixture of frozen chironomid larvae and commercial granulated shrimp food (Hikari Shrimp Cuisine). The perch were fed with living gammarids (a mixture of species tested). Light conditions were natural, not supported by any artificial lights.

### Experimental setup and test procedure

To check the effect of continuity of exposure to predation signals on prey physiology, we used four groups of adult gammarids from each species (mean size of gammarids: *D. villosus* 1.7 cm, *G. jazdzewskii* 1.1 cm). The first group received the predation cue once a day for a period of one week (the stimulus was added every day at the same time: 12:00 pm). The second group received the predation cue eight times a day, every 3 h, for a period of one week (start of the first exposure: 12:00 pm). The third and fourth groups were appropriate controls, receiving corresponding doses of pure water from a fishless tank. It was experimentally demonstrated the defence reaction of gammarids was induced by a 3-h old fish kairomone, but not by a 6-h old kairomone^24^. This indicates that the first group of the tested gammarids was affected by the predation cue periodically, whereas the second group was under the constant impact of the predator signal. Fifteen individuals of a single species were exposed together, forming one replicate (N = 12 per treatment per species). They were placed in a 1-L circular container (filled up to 0.7 l) made of white non-transparent plastic. All containers were constantly aerated and equipped with gravel bottom to be used as a shelter and tubes connected to a dosing pump (AQUA NOVA NMDP-5 JEBAO DP-5, Konojady, Poland). A single dose consisted of 35 ml of water pumped directly from a 40-L source tank containing either (1) two perch (10 cm in total length) or (2) conditioned tap water (with no fish). The source tanks were filled with tap water 7 days before the start of the experiment. Three days before the start of the experiment, the perch were placed in the fish source tank. From this moment until the end of the experiment, the fish were fed every day with the same gammarid species (20 individuals of *D. villosus* or 40 individuals of *G. jazdzewskii* to account for the difference in gammarid size) as those tested in the container receiving water from a given source tank. Water in the source tanks was constantly filtered and aerated. During the test, the test gammarids were fed daily with the same food as during the acclimation period (amount exceeded their daily demand). Each day at 4 pm, the volume of water necessary to keep the initial volume of 0.7-L constant was removed from each container. On this occasion, wastes and unconsumed food remnants were also removed. On the last day, 30 minutes after the last signal application in all the treatments, gammarids were gently removed from the containers, weighed to the nearest 0.1 mg with Radwag AS 110/C/2 laboratory scales (Radom, Poland), homogenised and frozen (−80 °C) until the determination of physiological markers.

### Response variables

Extracts for biochemical analyses were prepared from pooled specimens from each replicate to obtain sufficient amount of material^22^. Gammarids were homogenized in 5 ml of potassium phosphate buffer at pH 7.4 using a Potter homogenizer with a Teflon piston (200 rotations per min.) at 4 °C. After centrifugation at 12000 g for 10 min. at 4 °C, the supernatants were collected in Eppendorf tubes and stored at −80 °C until analysis. Then, the samples were used for determination of TAS, as well as TBARS, Hsp70 and glycogen concentrations.

TAS, as well as Hsp70 and glycogen concentrations were determined with commercial kits according to the manufacturers’ instructions (Sigma-Aldrich, Germany, catalogue number: CS0790-1KT; Biorbyt, United Kingdom, catalogue number orb397140; Sigma-Aldrich, Germany, catalogue number: MAK016-1KT, respectively). Colorimetric changes in the assay were detected using a multi-mode microplate reader Epoch 2 (BioTek Instruments, Inc., Winooski, UT, USA). The Total Antioxidant Status was expressed in mmol/ml, Hsp70 concentration as pg/ml and glycogen concentration as µg/ml.

TBARS concentration was determined according to Rice-Evans, Diplock & Symons^25^. For each sample, 1 ml of homogenate was added to 1 ml of 15% TCA (w/v) and 0.37% (w/v) TBA in 25 mM HCl. The samples were boiled for 10 minutes, then quickly cooled and immediately centrifuged for 5 minutes at 6500 g. Parallel, two blank samples were prepared: one without TBA and the other without the homogenate. All samples were subjected to the spectrophotometric analysis at 535 nm (Schimadzu UV-1800 spectrophotometer, Shimadzu Inc., Kyoto, Japan). Concentration of TBARS was expressed in nmol/g wet tissue.

### Statistical analyses

To analyse the effect of the predator presence and continuity of exposure to the predation cue (constant vs. periodic exposure) on biochemical markers in gammarids, we applied 2-way General Linear Models (GLM), separately for each species. To meet the GLM assumptions (normality was tested with a Shapiro-Wilk test and homoscedasticity with a Levene test), the data were log-transformed when necessary. Significant terms were further explored using sequential Bonferroni corrected LSD post-hoc tests. The analyses were carried out using SPSS 25.0 package (IBM Inc.).

## Results

**Figure 1:**
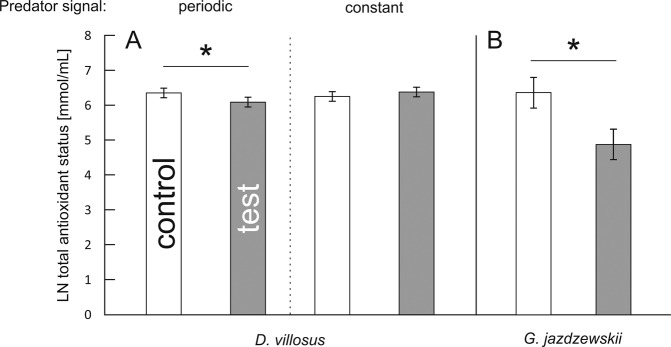
(**A,B**) Effect of continuity of predation risk on Total Antioxidant Status (TAS) in two gammarid species. Presented values are means predicted by the GLM models for each species separately. Asterisks indicate significant differences in TAS level between predator-stressed and corresponding control individuals.

The level of TAS in *D. villosus* depended on a 2-way interaction between continuity of predation risk and predator presence (Table 1A). *D. villosus* exposed to the periodic predation risk demonstrated lower TAS compared to the control individuals (Fig. 1A). In *G. jazdzewskii*, predation risk caused a significant reduction in TAS irrespective of its continuity, as shown by a significant main effect of predator presence (Table 2A, Fig. 1B).

**Table 1:** The 2-way General Linear Model to test the effect of the predator presence and predation risk continuity on the total antioxidant status (TAS), concentration of thiobarbituric acid reactive substances (TBARS), concentration of heat shock proteins (Hsp70) and concentration of glycogen in *D. villosus*.

	Dependent variable	Effect	df	MS	F	P
A	TAS	Predator presence	1	0.045	0.979	0.329
		Predation risk continuity	1	0.087	1.878	0.179
		Interaction	1	0.383	8.278	0.007
		Error	36			
B	TBARS concentration	Predator presence	1	0.199	4.322	0.044
		Predation risk continuity	1	0.082	1.794	0.188
		Interaction	1	0.030	0.647	0.426
		Error	39			
C	Hsp70 concentration	Predator presence	1	0.062	3.44	0.072
		Predation risk continuity	1	0.224	12.401	0.001
		Interaction	1	0.136	7.531	0.010
		Error	35	0.018		
D	Glycogen concentration	Predator presence	1	0.369	1.722	0.199
		Predation risk continuity	1	0.597	2.789	0.105
		Interaction	1	0.239	1.115	0.299
		Error	32	0.214

**Table 2:** The 2-way General Linear Model to test the effect of the predator presence and predation risk continuity on the total antioxidant status (TAS), concentration of thiobarbituric acid reactive substances (TBARS), concentration of heat shock proteins (Hsp70) and concentration of glycogen in *G. jazdzewskii*.

	Dependent variable	Effect	df	MS	F	P
A	TAS	Predator presence	1	21.966	23.319	<0.001
		Predation risk continuity	1	29.671	31.499	<0.001
		Interaction	1	1.391	1.477	0.232
		Error	36	0.942		
B	TBARS concentration	Predator presence	1	0.004	0.108	0.745
		Predation risk continuity	1	1.318	33.57	<0.001
		Interaction	1	0.352	8.979	0.005
		Error	42	0.039		
C	HSP 70 concentration	Predator presence	1	0.152	1.628	0.212
		Predation risk continuity	1	1.655	17.671	<0.001
		Interaction	1	0.467	4.984	0.033
		Error	30	0.094		
D	Glycogen concentration	Predator presence	1	0.001	0.003	0.958
		Predation risk continuity	1	4.227	12.578	0.001
		Interaction	1	1.687	5.021	0.032
		Error	32	0.336		

**Figure 2:**
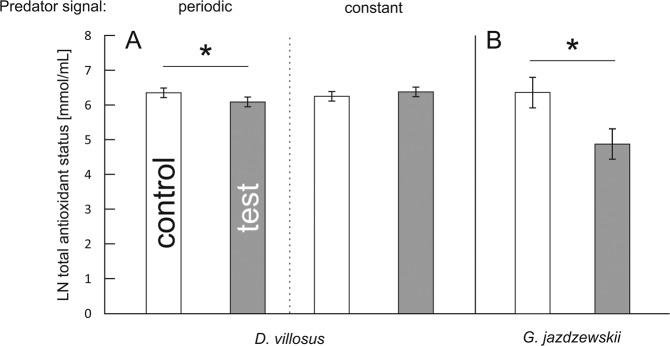
(**A,B**) Effect of continuity of predation risk on lipid oxidative damage (TBARS) in two gammarid species. Presented values are means predicted by the GLM models for each species separately. Asterisks indicate significant differences in TBARS level between predator-stressed and corresponding control individuals.

Individuals of *D. villosus* exposed to predators demonstrated lower TBARS concentration compared to their respective controls, as shown by a significant main effect of predator presence (Table 1B, Fig. 2A). However, it should be noted that this result was only marginally significant. In *G. jazdzewskii*, TBARS concentration depended on a 2-way interaction between continuity of predation risk and predator presence (Table 2B). Individuals exposed to the periodic predation risk showed a lower TBARS concentration than control gammarids (Fig. 2B).

**Figure 3:**
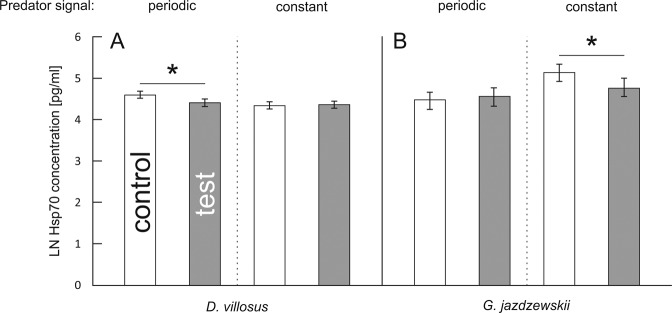
(**A,B**) Effect of continuity of predation risk on the concentration of heat shock protein (Hsp70) in two gammarid species. Presented values are means predicted by the GLM models for each species separately. Asterisks indicate significant differences in Hsp70 concentration between predator-stressed and corresponding control individuals.

Hsp70 concentrations in both species depended on 2-way interactions between continuity of predation risk and predator presence (Tables 1C and 2C). Compared to respective controls, this parameter was reduced under periodic (*D. villosus*, Fig. 3A) or constant (*G. jazdzewskii*, Fig. 3B) predation risk.

**Figure 4:**
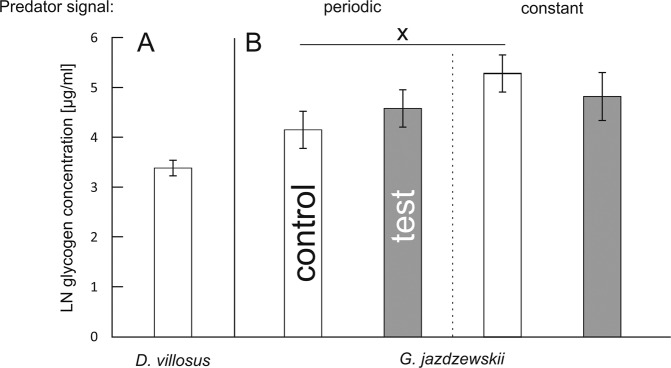
(**A,B**) Effect of continuity of predation risk on glycogen concentration in two gammarid species. Presented values are means predicted by the GLM models for each species separately. “X” marks indicate significant differences in glycogen concentration between periodic and constant exposure treatments.

Glycogen concentration in *D. villosus* was not affected by any experimental factor (Table 1D, Fig. 4A), while in *G. jazdzewskii* it depended on a 2-way interaction between predator presence and predation risk continuity (Table 2D). Nevertheless, according to the post-hoc comparisons, predator presence did not affect significantly the glycogen concentration in any experimental treatment (Fig. 4B).

## Discussion

Despite the growing attention of ecologists, the role of chronic predation risk on prey functioning, including its physiological aspects, is still unclear^26,27^. So far, it has been demonstrated that chronic predation risk (defined as 1 stress episode per day during 7 days) induces an increase in oxygen consumption, causing the higher synthesis of reactive oxygen species (ROS) responsible for reduction of defence capabilities due to oxidative damage^14,23^. On the other hand, it was shown that stimulation of prey by predation cues increases its survival rate in the predator presence^21^ and leads to improvement of escape abilities^28,29^. Moreover, other studies demonstrated that prey individuals exhibited symptoms of oxidative stress only under an initial contact with the predation cue (after 35 minutes of exposure) while a prolonged and constant contact with the cue (7 days of constant exposure) resulted in the habituation to the stressor^22^. These examples suggest that not only the duration of exposure, but also the continuity of predation risk (i.e. the frequency of stress episodes during the long-term exposure) may determine its final physiological consequences. Here we experimentally confirmed that the continuity of predation risk is a crucial factor determining long term responses of prey. All the response variables tested (except glycogen concentration) were affected by the predator presence, however the quality of these modifications depended on the continuity of the predation risk and/or prey species.

Contrary to our prediction, the repeating stimulation by predator kairomones once a day during one week (periodic predation risk) did not generate a significantly higher damage than the constant stimulation during the same period. After one week of exposure to the periodic predation risk, both species exhibited a lower total antioxidant status compared to control individuals. However, in contrast to previous studies^13,23^, the reduction in the antioxidant defence potential was not related to the higher level of oxidative damage (TBARS concentration). Actually, individuals of both species exposed to the periodic predation risk showed a lower level of lipid peroxidation than in the control treatments. A similar response was also shown by *D. villosus* exposed to the constant predation risk treatment, though it was visually weaker than during the periodic risk exposure. So far, it has been demonstrated that predation risk forces prey to increase their metabolic rate, which maximizes the performance of anti-predator defence reactions^30^. However, in consequence of the higher ROS synthesis due to increased metabolism, an organism is also forced to activate its antioxidant defence systems^12^. When the antioxidant defence is insufficient, prey suffer oxidative stress due to devastating activities of ROS^23,31^. As the maintenance of the high level of antioxidant defences is extremely costly, the time of their increased activation is limited^32^. Moreover, some authors suggest that under predation risk prey can relocate energy from antioxidant defence into responses directly related to antipredator defence mechanisms^10,33^. Therefore a prolonged exposure to predation risk may result in oxidative damage due to the reduction in antioxidant defence efficiency. Here, contrary to our main hypothesis, we demonstrated that regular but interrupted (i.e. periodic) stimulation of prey with the predation cue may generate a reverse effect than it has been shown so far. A lower level of lipid peroxidation indicates either the lower pressure of ROS or the higher efficiency of antioxidant defence systems. As the periodic predation risk treatment was accompanied by the lower level of total antioxidant capacity (compared to control individuals), the former explanation is more likely. The lower pressure of ROS is also associated with the lower damage of other key biological molecules, such as DNA or proteins^31^. Reduction in the ROS synthesis could be explained by the reduction in metabolic rate, which was observed in freshwater mussels after the prolonged exposure to the predator cue^34^. However, gammarids were shown to increase their metabolism when exposed to acute as well as constant and prolonged predator presence^22^. Nevertheless, our present findings as well as some other studies (unpublished data) suggest the picture is much more complex and gammarids may either reduce or increase their metabolic rate in the predator presence and their reaction is dependent on environmental conditions. As metabolic rate is directly related to animal movement, ROS synthesis could be also controlled by behavioural modification^35^. Movement reduction is a common response to predator presence^36^, therefore, apart from its beneficial effect on prey survival, it could also help save energy resources by decreasing the metabolic rate.

Contrary to the previous research focused on predation risk, both species showed down-regulation of the heat shock protein concentration when regularly stimulated by the predator cue (under periodic or constant exposure in *D. villosus* and *G. jazdzewskii*, respectively). The role of heat shock proteins is associated with sustaining homeostasis under challenging conditions^37^, acceleration of the immune system during injuries^38^ and driving fight or flight response^39^. So far, it has been demonstrated that both acute and chronic periodic predation risk up-regulate the synthesis of heat shock proteins^13,22^. On the other hand, it has also been shown that the synthesis of heat shock proteins under prolonged and continuous stimulation by the predator cue is lower than during the acute predation exposure^22^. Moreover, in some cases the level of stress proteins is not correlated with predation risk^40^. The heat shock protein reduction observed in our study potentially exposes prey to the disturbance of homeostasis, however, the TBARS level was not increased in any experimental treatment, suggesting the lack of negative consequences of this physiological modification.

Synthesis as well as maintenance of cellular defence systems are extremely costly due to their catabolic function, therefore, they may contribute to the growth reduction under stressful conditions^37,41^. Predator presence generates significant disturbances in energy acquisition due to behavioural modification^1^ and energy storage by physiological adjustments^18^, therefore the observed reduction in costly defence mechanisms could help in maintaining energy resources intact as long as possible. In the present study, the level of glycogen in both species was not affected by predator presence independently of the continuity of predation risk, indicating the effectiveness of their energy saving strategy. Sustaining the constant amount of energy storage under predation risk could be supported by physiological adjustments. For example, undisturbed concentration of glycogen accompanied by lower ROS pressure can be the final result of the reduction in metabolic rate (as suggested by the lower level of oxidative damages). The lower metabolic rate can result in the lower synthesis of ROS and, in consequence, lower activity of energetically costly antioxidant defences, giving the opportunity to accumulate storage materials. The last possible physiological adjustment to maintain the glycogen level untouched is the increased digestive efficiency induced by the predator presence. So far, such a mechanism has been observed in hornworm caterpillars^19^ and damselfly larvae^42^, but it has never been tested in gammarids. However, we already know that *D. villosus* does not reduce it consumption and growth rate under predation risk, particularly if food resources are in its direct proximity^1^.

Some differences between the test species were noted, for example the reduction in TBARS of *D. villosus* occurred independently of the type of predation risk (constant vs. periodic), whereas *G. jazdzewskii* showed such a response only when periodically stimulated by the predator cue. *Dikerogammarus villosus* exhibits one of the highest survival rates among gammarids when experimentally exposed to predators, indicating the effectiveness of its anti-predator defences^43–45^. Compared to G. jazdzewskii, *D. villosus* is more armoured, less active, efficiently uses hard substrata as shelters and is able to form aggregations efficiently reducing predation risk^44,46^. A large part of the *D. villosus* defence strategy is constitutive, rather than directly induced by predator presence^47^. However, despite differences in their antipredator strategy and physiological adjustments to chronic predation risk, the final effects of the prolonged predator pressure, such as the lack of oxidative stress and decrease in glycogen level, seem similar between the test species.

Previous studies showed that the prolonged predator pressure generated a significant modification in prey physiology, including cellular damage^23^ and reduction in glycogen concentration^18^, as well as the increase in defence abilities^21^. Here we demonstrated that prey organisms reconfigure their physiology to keep their homeostasis and reduce potential fitness costs when face prolonged predator pressure. Moreover, these physiological adjustments depend on predation risk continuity. Furthermore, our results showed that the prolonged predator pressure might indirectly generate a reduction in oxidative damage in prey organisms, which, along with the lack of reduction in glycogen content, shows that they can thrive even under high predator pressure and reorganize their physiological processes to avoid negative consequences of non-consumptive predator effects. Further studies on a higher number of species are needed to confirm whether the interspecific differences in physiological responses observed in our study are related to the invasive potential and/or nature (constitutive vs. induced) of defence strategies exhibited by various species.
