# Author Correction: Continuity of chronic predation risk determines changes in prey physiology

**DOI:** 10.1038/s41598-021-88181-z

**Published:** 2021-04-13

**Authors:** Łukasz Jermacz, Hanna Kletkiewicz, Anna Nowakowska, Anna Dzierżyńska-Białończyk, Maciej Klimiuk, Jarosław Kobak

**Affiliations:** 1 Nicolaus Copernicus University, Faculty of Biology and Veterinary Sciences, Department of Invertebrate Zoology and Parasitology, Lwowska 1, 87-100 Toruń, Poland; 2 Nicolaus Copernicus University, Faculty of Biology and Veterinary Sciences, Department of Ecology and Biogeography, Lwowska 1, 87-100 Toruń, Poland; 3 Nicolaus Copernicus University, Faculty of Biology and Veterinary Sciences, Department of Animal Physiology, Lwowska 1, 87-100 Toruń, Poland

Correction to: *Scientific Reports* 10.1038/s41598-020-64000-9, Published online 24 April 2020

In this Article, Figure 2 is a duplication of Figure 1. The correct Figure 2 appears below as Figure 1.

**Figure 1:**
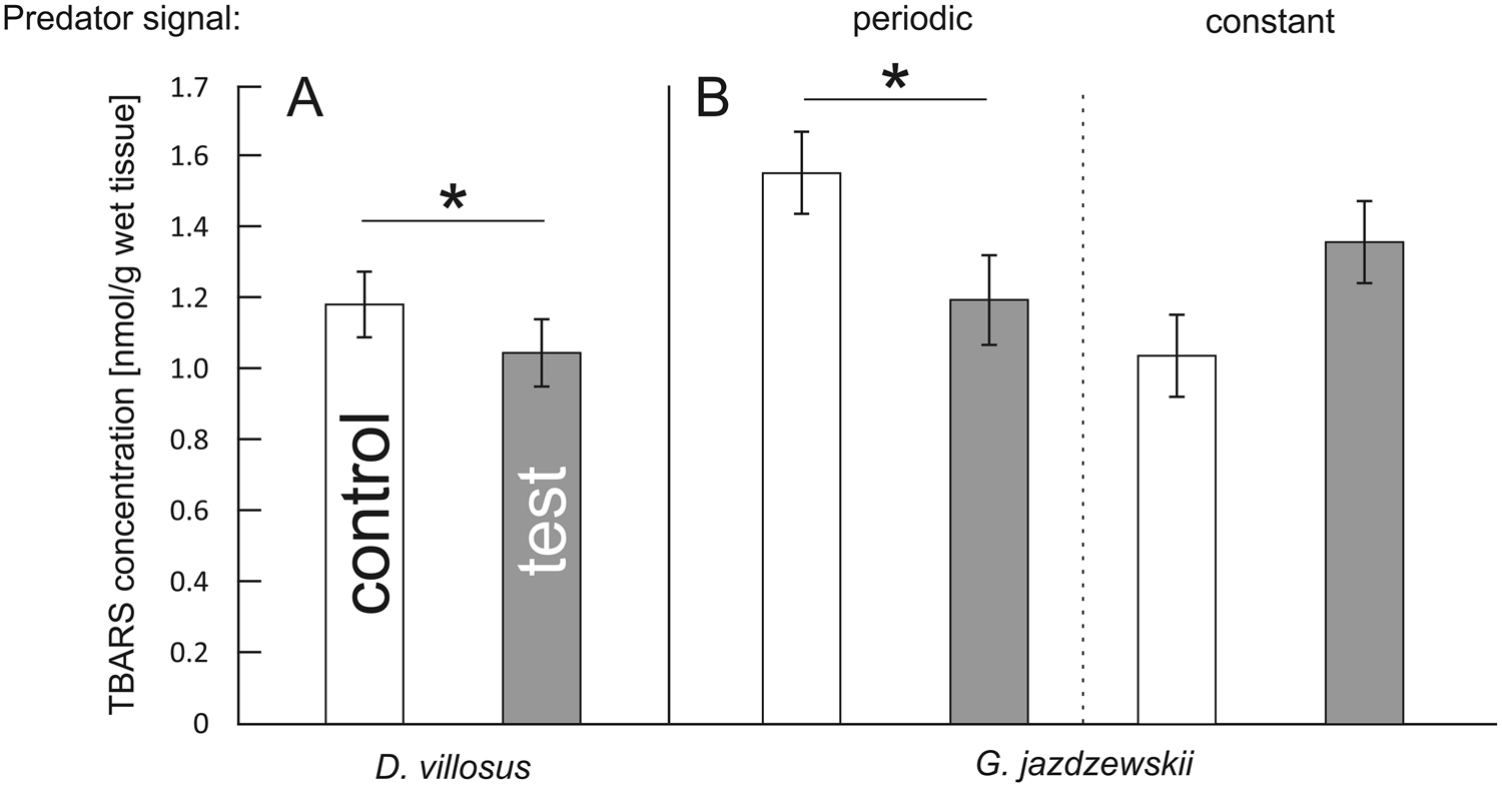
A correct version of the original Figure 2.

